# Sensitive detection of miRNA by using hybridization chain reaction coupled with positively charged gold nanoparticles

**DOI:** 10.1038/srep32358

**Published:** 2016-08-31

**Authors:** Xiangmin Miao, Xue Ning, Zongbing Li, Zhiyuan Cheng

**Affiliations:** 1 School of Life Science, Jiangsu Normal University, Xuzhou 221116, PR China; 2 KeWen College, JiangSu Normal University, Xuzhou 221116, PR China

## Abstract

Positively charged gold nanoparticles (+)AuNPs can adsorb onto the negatively charged surface of single-stranded DNA (ssDNA) or double-stranded DNA (dsDNA). Herein, long-range dsDNA polymers could form based on the hybridization chain reaction (HCR) of two hairpin probes (H_1_ and H_2_) by using miRNA-21 as an initiator. (+)AuNPs could adsorb onto the negatively charged surface of such long-range dsDNA polymers based on the electrostatic adsorption, which directly resulted in the precipitation of (+)AuNPs and the decrease of (+)AuNPs absorption spectra. Under optimal conditions, miRNA-21 detection could be realized in the range of 20 pM-10 nM with a detection limit of 6.8 pM. In addition, (+)AuNPs used here are much more stable than commonly used negatively charged gold nanoparticles ((−)AuNPs) in mixed solution that contained salt, protein or other metal ions. Importantly, the assay could realize the detection of miRNA in human serum samples.

MicroRNA (miRNA) plays significant regulatory role in a diverse range of biologicals processes including cell proliferation, apoptosis and death^1^. The aberrant expressions of miRNA in serum or plasma are related to various diseases such as cancers, viral infections, neurological diseases and Alzheimer’s disease^2^,^3^. For instance, high level of miRNA-21 is correlated with human cancers including breast cancer, pancreatic cancer, colorectal cancer, etc.^4^,^5^,^6^. Thus, developing methodologies for the detection of miRNAs are of great importance in clinical disease diagnosis, gene therapy and discovery of new anticancer drugs^7^. Traditional methods for miRNA detection mainly use the real-time reverse transcription polymerase chain reaction (RT-PCR)^8^, northern blotting^9^, miRNA array technology^10^ and different isothermal amplification techniques^11^,^12^,^13^,^14^,^15^. Recently, a number of novel sensing approaches have been developed by using colorimetry^16^,^17^,^18^,^19^, fluorescence^20^,^21^,^22^,^23^,^24^,^25^, electrochemiluminescence^26^,^27^, electrochemistry^28^,^29^,^30^,^31^, and surface plasmon resonance (SPR)^32^,^33^ as detection platform. Thereinto, colorimetric based methods have attracted great attention because of the merits of them including rapidness, simplicity and low cost^34^.

The key point in colorimetric-based bioanalysis is to improve the sensitivity of such proposals. Recently, numerous amplification strategies including rolling circle amplification^35^,^36^,^37^, enzymatic signal amplification^38^,^39^,^40^ and strand displacement assay^41^ have developed. However, these amplified strategies suffered from some weaknesses including complex process, high cost and possible false response because of the utilization of enzyme^42^. Therefore, hybridization chain reaction (HCR), as one type of enzyme-free amplification strategies, can be used as an ideal candidate, which is driven by the self-assembly of two stable species of DNA hairpins^43^ and can also exhibit the advantages of simple operation, low background, low cost and PCR-like sensitivity^44^.

Due to the unique optical properties and high molar extinction coefficient of gold nanoparticles (AuNPs)^45^, they have been widely used for the preparation of colorimetric biosensors, based on detecting the color change of AuNPs from red to blue (assembly) or from blue to red (disassembly) induced by the distance change of particles^46^,^47^,^48^. AuNPs used in such sensing strategies are mainly negatively charged and the disperse states of them can be significantly affected in complex systems that contained salt, DNA, proteins or other ions, which limited the application of them in biological and complex systems assay. Recently, researchers reported several novel colorimetric-based strategies by using positively charged AuNPs ((+)AuNPs) as signal probe based on the adsorption of them to negatively charged surface of DNA^49^,^50^. Such label-free methods are relatively simple and low-cost, without needing the modification of substrate with AuNPs.

Here we developed for the first time a versatile and sensitive label-free sensor for miRNA-21 detection based on the precipitation of (+)AuNPs, and such precipitation happened automatically along with the increase of miRNA-21 concentration, which without needing an extra magnet just as the literatures^51^,^52^,^53^. Meantime, such proposed strategy avoided the lengthy conjugation process between DNA and AuNPs. In addition, experiments testified that (+)AuNPs used here are much more stable than negatively charged AuNPs in mixed systems containing salt, DNA, protein or metal ions (See result and discussion part), making our proposed method more tolerant to the sensing environments. Moreover, the assay contributed the new application of (+)AuNPs and also expanded the scope of miRNA based sensing. Finally, a satisfactory result obtained while detecting miRNA-21 in human serum samples.

## Results and Discussion

### Detection mechanism of miRNA-21

**Figure 1:**
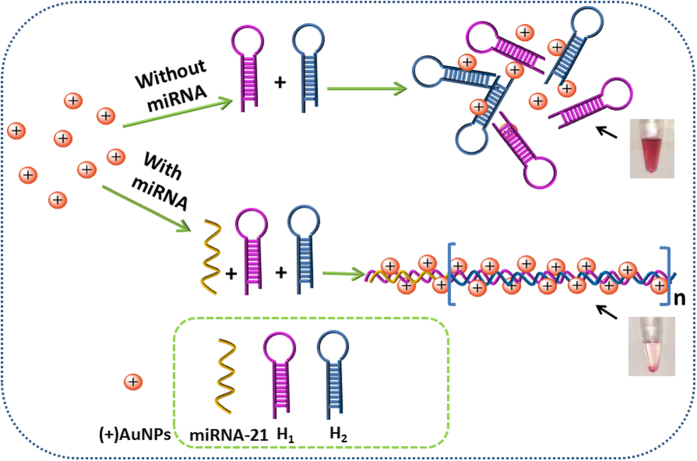
Scheme of miRNA-21 detection based on the precipitation of DNA-AuNPs nanostructure.

To construct such a sensor, HCR happened between two hairpin probes (H_1_ and H_2_) by using the target miRNA-21 as an initiator to form long-range dsDNA polymers. Then, numerous (+)AuNPs could adsorb onto the surface of such long-range dsDNA polymers based on the electrostatic adsorption, and accordingly resulted in the precipitation of them because of the increase of their gravity. Meantime, more HCR products could be formed along with the increase of miRNA concentration, which directly induced the precipitation of (+)AuNPs more easily as a result. At last, miRNA-21 could be detected facilely based on detecting the concentration of (+)AuNPs in supernatant by using the simple UV-vis spectrum method (Fig. 1).

### Characterizations of (+)AuNPs

**Figure 2:**
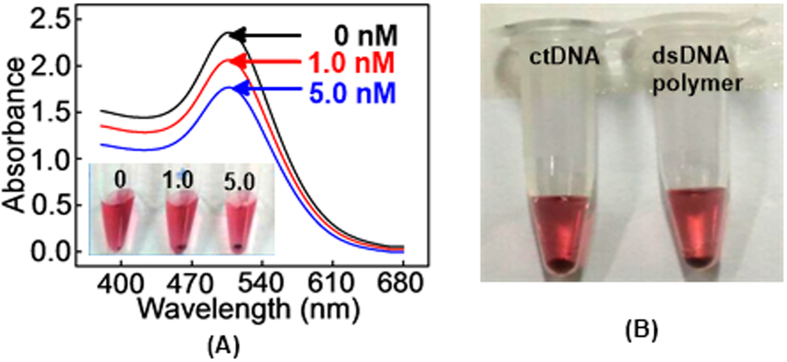
(**A**) UV-vis absorption spectra of (+)AuNPs with H_1_/H_2_ mixture (300 nM, 1:1 ratio) (a), (a)+1.0 nM of target miRNA-21 (b), and (a)+5.0 nM target of miRNA-21 (c) (inset: corresponding photographs of (+)AuNPs); (**B**) Photographs of (+)AuNPs after the incubation of them with ctDNA (a) and long-range dsDNA polymers (b).

UV-vis spectrum of (+)AuNPs in supernatant were performed in order to investigate the characteristics of the proposed method. Figure 2A showed the UV-vis spectrum and the corresponding photographs of (+)AuNPs. In the absence of target miRNA-21, a high adsorption spectra of (+)AuNPs was observed at 510 nm (a), and no precipitation was found (inset, a). However, in the presence of 1.0 and 5.0 nM of target miRNA-21 (b and c), the adsorption spectra of (+)AuNPs at 510 nm decreased obviously, accompanying with the precipitation of them (inset, b and c). Such results directly illustrated that HCR happened between H_1_ and H_2_ to form long-range dsDNA polymers for the precipitation of (+)AuNPs. The reason for such precipitation might be due to the increased specific gravity of DNA-AuNPs nanostructure. Literatures have reported that the change of AuNPs color from orange red to blue was mainly because of the increase of particle size or the massive aggregate of AuNPs^54^,^55^. Herein, the color of (+)AuNPs did not change only with a precipitation. Thus, it could be supposed that the precipitation of (+)AuNPs was mainly due to the adsorption of them with DNA strands^56^ but not the production of massive aggregates.

Meantime, calf thymus DNA (ctDNA, dsDNA) was selected to further prove the adsorption properties of (+)AuNPs on negatively charged dsDNA strands. As seen in Fig. 2B (a), (+)AuNPs precipitation appeared upon the incubation of them with ctDNA (0.2 mg/mL), which was similar to the phenomenon of that (+)AuNPs were incubated with long-range dsDNA polymers (b). Such results further illustrated that the precipitation of (+)AuNPs was based on the adsorption of them with long-range dsDNA polymers.

**Figure 3:**
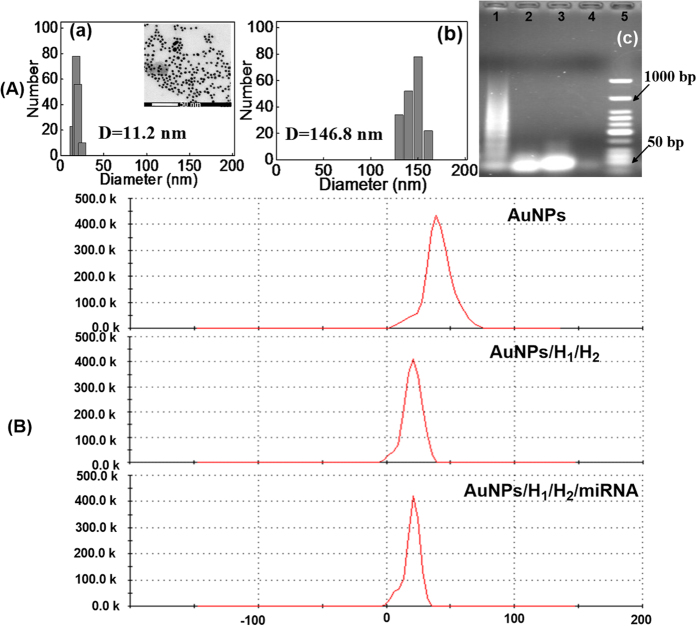
(**A**) DLS images of (a) (+)AuNPs (insert a: TEM images of (+)AuNPs), (b) after the incubation of (+)AuNPs with H_1_/H_2_ mixture (300 nM, 1:1 ratio) and 10 nM target miRNA-21, and (c) gel electrophoresis for DNA and RNA stands (lane 1: 1.0 μM miRNA-21 reacted with 1.0 μM of H_1_/H_2_ mixture, lane 2: 1.0 μM of H_1_/H_2_ mixture, lane 3: 1.0 μM of H_1_, lane 4: 1.0 μM miRNA-21 and lane 5: DNA marker); (**B**) Zeta potential analysis of (+)AuNPs (a), (+)AuNPs after incubating with H_1_/H_2_ mixture (300 nM, 1:1 ratio) in the absence (b) and presence of 5.0 nM target miRNA-21 (c).

The total size of (+)AuNPs increased after the incubation of them with long-range dsDNA polymers. As shown in Fig. 3A, the average hydrodynamic diameter of (+)AuNPs detected from dynamic light scattering (DLS) was 11.2 nm (a), which was bigger than the TEM results (insert a) because of that the DLS analysis measured the hydrodynamic radius while TEM analysis provided a more precise measurement of the hard AuNP core^57^. Then, upon the incubation of (+)AuNPs with the long-range dsDNA polymers formed between 300 nM of H_1_/H_2_ and 5.0 nM of target miRNA-21, (+)AuNPs assembly and precipitation happened and the average hydrodynamic diameter of (+)AuNPs increased to 146.8 nm (b). Such results were in good agreement with the UV-vis absorption spectra results in Fig. 2A.

HCR is a critical factor for constructing such a sensor. Thus, gel electrophoresis was conducted to monitor the happen of HCR between H_1_ and H_2_ by using the target miRNA-21 as an initiator. As shown in Fig. 3A(c), the base number of 1.0 μM miRNA-21 was about 25 nt (lane 4) while that for 1.0 μM of H_1_ (lane 3), 1.0 μM of H_1_ and H_2_ mixture (lane 2) were about 50 nt, which proved that no HCR happened between H_1_ and H_2_ in the absence of target miRNA-21. However, obvious smears obtained after the incubation of miRNA-21 (1.0 μM) with H_1_ and H_2_ mixture (1.0 μM, 1:1 ratio) (lane 1). Such results obviously proved the happen of HCR, and the smears might be attributed to the difference of dsDNA polymers in length, which was similar to other literatures^58^,^59^,^60^.

Moreover, the zeta potential analysis of (+)AuNPs was also constructed by using DLS to investigate the charge reduction of (+)AuNPs before and after the incubation of them with long-range dsDNA polymers. As seen in Fig. 3B, the zeta potential of (+)AuNPs was +39.8 mV (a) while it reduced to +15.1 V after the incubation of it with H_1_ and H_2_ mixture in the absence of miRNA-21, which mainly due to the neutralization of (+)AuNPs charge by negatively charged H_1_ and H_2_ DNA strands (b). Then, the zeta potential reduced to +10.1 V (c) upon the incubation of (+)AuNPs with long-range dsDNA polymers formed between 300 nM of H_1_/H_2_ and 5.0 nM of target miRNA-21. Such reduction of (+)AuNPs charge mainly due to the effective adsorption of them to negatively charged long-range dsDNA polymers.

### Optimization of the experimental conditions

**Figure 4:**
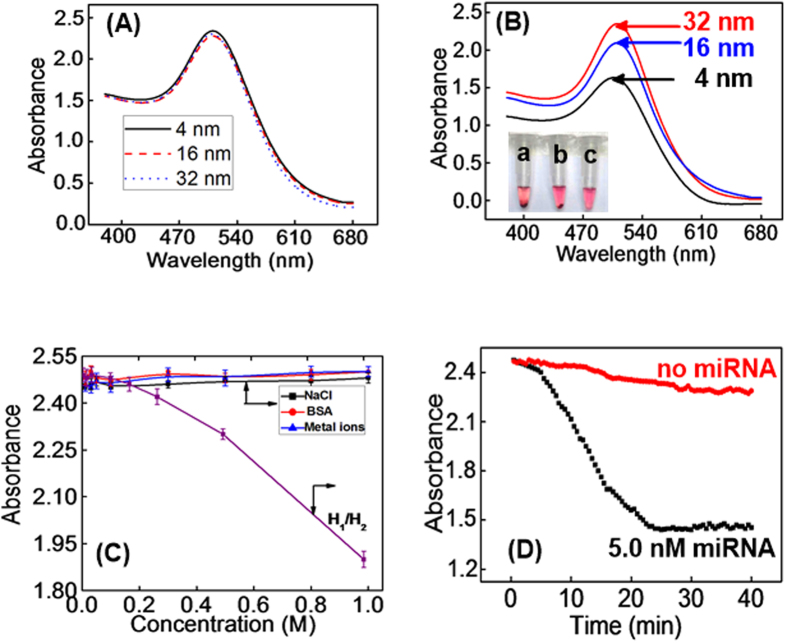
Absorption spectra of (+)AuNPs with different sizes ((a) 4, (b) 16, and (c) 32 nm) before (**A**) and after (**B**) the incubation of them with H_1_/H_2_ mixture (300 nM, 1:1 ratio) in the presence of 5.0 nM miRNA-21 (inset of Fig. 3B: corresponding precipitation of (+)AuNPs); (**C**) The effect of increasing concentration of NaCl (a), BSA (b) and metal ions (c) from 0 to 1.0 M, and increasing concentration of H_1_ and H_2_ mixture from 0 to 3.0 μM (d) to the stability of (+)AuNPs; (**D**) Effect of the incubation time between (+)AuNPs and H_1_/H_2_ mixture (300 nM, 1:1 ratio) in the absence and presence of 5.0 nM miRNA-21.

To investigate the size effect of (+)AuNPs on the performance of the sensor, three types of (+)AuNPs samples (4 nm, 16 nm and 32 nm) were prepared and the UV-vis absorption spectra of them were similar (Fig. 4A). Then, three types of (+)AuNPs were incubated with H_1_ and H_2_ mixture (300 nM, 1:1 ratio) respectively in the presence of 5.0 nM of target miRNA-21. As shown in inset of Fig. 4B, the degree of (+)AuNPs precipitation was decreased along with the increase of (+)AuNPs size (a: 4 nm, b: 16 nm and c: 32 nm), followed by the increase of (+)AuNPs absorption spectra (Fig. 4B). Such phenomena indicated that 4 nm of (+)AuNPs in favor of the precipitation of AuNPs-DNA nanostructure. Thus, 4 nm of (+)AuNPs was selected as the signal probe for all of the experiments.

To prove the stability of (+)AuNPs in mixed solution, the effect of salt (NaCl as example), protein (BSA as example), metal ions (K^+^, Ca^2+^ and Mg^2+^ as example) and DNA probes concentration was investigated. As shown in Fig. 4C, no obvious change of (+)AuNPs absorption spectra appeared along with the increase of NaCl (a), BSA (b) and metal ions (c) concentration from 0 to 1.0 M, respectively. Such results certainly indicated that (+)AuNPs are highly stable in mixed environments. However, if the concentration of H_1_ and H_2_ was higher than 300 nM, (+)AuNPs precipitation happened because of the absorption of them with DNA strands. Thus, 300 nM of H_1_/H_2_ was used in this sensor.

Moreover, the incubation time of (+)AuNPs with H_1_ and H_2_ mixture (300 nM) in the absence and presence of target miRNA-21 was investigated in Fig. 4D. The adsorption spectra of (+)AuNPs had slight change against the incubation time in the absence of miRNA-21 (a), which mainly due to the adsorption of a small number of (+)AuNPs with H_1_ and H_2_ strands. However, in the presence of 5.0 nM miRNA-21, the adsorption spectra of (+)AuNPs gradually decreased with increasing incubation time over the range from 0–25 min (b), and then reached a plateau. Results indicated that 25 min was enough for the effectively adsorption of (+)AuNPs with negatively charged dsDNA polymers. Thus, 25 min was selected as the optimum incubation time.

### Performance of the sensor

**Figure 5:**
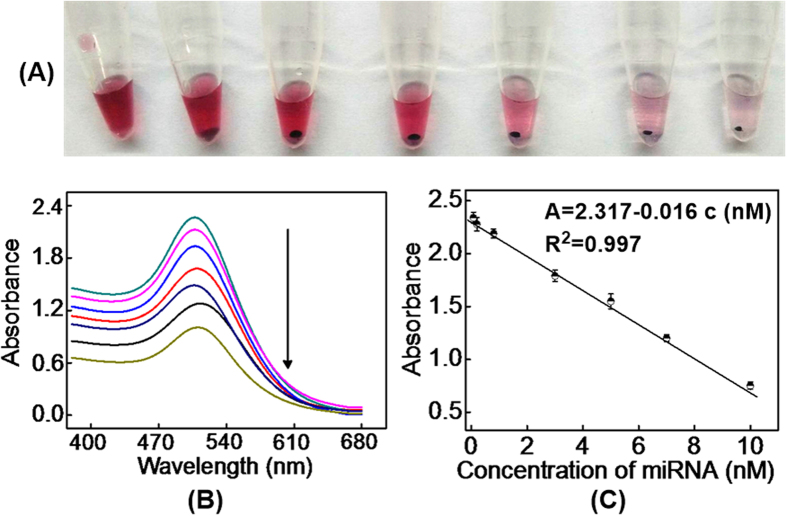
(**A**) The photographs (**A**) and the UV-vis absorption spectra (**B**) of (+)AuNPs after the incubation of them with H_1_/H_2_ mixture (300 nM, 1:1 ratio) in the presence of different concentration of target miRNA-21; (**C**) The calibration curve for target miRNA-21 detection corresponding to (**B**).

Properties of the proposed method was investigated based on detecting the change of (+)AuNPs absorption spectra at 510 nm upon the incubation of them with H_1_ and H_2_ mixture (300 nM, 1:1 ratio) in the presence of different concentrations of target miRNA-21. As shown in Fig. 5A, (+)AuNPs precipitation increased along with the increase of target miRNA-21 concentration, and followed by the decrease of (+)AuNPs absorption spectra at 510 nm (Fig. 5B). A linear range of miRNA-21 concentration from 20 pM to 10 nM was obtained with a detection limit of 6.8 pM (containing 1.7 × 10^6^/μL copies) recorded using the 3σ method (Fig. 5C). The linear regression equation was A = 2.317-0.016 c (c: nM, R^2^ = 0.997). Moreover, the detection limit of our assay could compare with some nanoparticle-based optical methods because of the HCR signal amplification of our method^18^,^61^,^62^. Meantime, our method was simple, without needing the using of enzyme and expensive instrument, which made our sensor more suitable for bioanalysis than other methods by using enzyme (Table 1)^16^,^63^.

### Selectivity of the sensor

**Figure 6:**
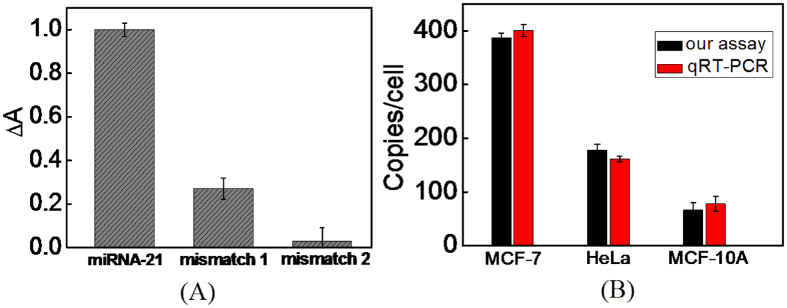
(**A**) Selectivity of the sensor for 5.0 nM of target miRNA-21 against 5.0 nM of mismatch 1 and mismatch 2; (**B**) miRNA-21 detection in different cancer lines including MCF-7, Hela and MCF-10A by using the methods of qRT-PCR (red bars) and the presented method (black bars), respectively.

To investigate the sequence specificity of the assay, we designed two types of mismatch RNA strands, named as mismatch 1 and mismatch 2, respectively. One U base in mismatch 1 was replaced by C while two U bases in mismatch 2 were replaced by CG. The selectivity of the sensor for miRNA-21 detection was investigated by monitoring the UV-vis absorption spectra change (ΔA) of (+)AuNPs after incubating them with H_1_ and H_2_ mixture in the presence of miRNA-21, mismatch 1 and mismatch 2, respectively. As shown in Fig. 6A, the ΔA of (+)AuNPs induced upon the addition of 5.0 nM of miRNA-21 was 1.0, while they were 0.27 and 0.03 for mismatch 1 and mismatch 2. Such results directly indicated that the proposed sensor has high selectivity for miRNA detection.

### Application of the sensor in real samples

To evaluate the application of the sensor for miRNA-21 detection in real samples, human serum samples were collected from Xuzhou Central Hospital. Then, the recovery experiments were constructed using standard addition method by adding 50 pM, 500 pM and 5.0 nM of miRNA-21 to human serum samples. As shown in Table 2 a good recovery in the range of 97.8–101.3% was obtained, and the relative standard deviation (RSD) was below 4.63%. Meantime, the concentrations of miRNA-21 in three human cancer cell lines were detected by using our assay and qRT-PCR method. As shown in Fig. 6B, the detection results obtained by using our assay were consistent with those of the qRT-PCR method, and each MCF-7 cell contains ~382 copies of miRNA-21, which was similar to the result of the literature^64^. Such results indicated that the proposed sensing platform has a potential application for the detection of miRNA-21 in complex biological samples.

In conclusion, a versatile and label-free sensor for target miRNA-21 detection was developed based on the precipitation of (+)AuNPs. Combining (+)AuNPs with HCR dual signal amplification, a detection limit down to 6.8 pM was obtained for target miRNA-21, which was much more sensitive compared with other colorimetric based methods. In addition, such visual based detection method was simple and rapid, without needing the label of DNA strands and using any experiment instruments. Moreover, (+)AuNPs, used in such strategy, has good stability in mixed solution that contained salt, protein or other metal ions. These advantages make our method more convenient for the application of biochemical and biomolecules detection.

## Methods

### Materials and apparatus

DNA sequences used in the experiments were synthesized by Shanghai Sangon Biotech Co. Ltd (Shanghai, China). MiRNA strands were synthesized by TaKaRa (Dalian, China), and were maintained at −20 °C before using. Calf thymus DNA (ctDNA) was purchased from Sigma Chemical Co. (St. Louis, MO, USA). The sequences of DNA and miRNA were shown in Table 3. The 22-nt bases of H_1_ at the 5′ end (italic) were used to hybridize with miRNA-21, and 24-nt bases of H_1_ at the 3′ end (blue underline) were used to hybridize with 24-nt bases of H_2_ at the 3′ end (red underline). Then, the 22-nt bases of H_2_ at the 5′ end could hybridize with another H_1_ strands, and further induced the occurrence of HCR between H_1_ and H_2_. Before using, H_1_ and H_2_ were heated to 95 °C and stayed for 5 min, then allowed to cool to room temperature in one hour, and the anneal product was stored in a refrigerator at 4 °C before use. Cetyltrimethyl ammonium bromide (CTAB), chloroauric acid (HAuCl_4_) and sodium borohydride (NaBH_4_) were purchased from Aladdin Biotech CO. Ltd. (Beijing, China). Ultra-pure water was obtained from Heal Force Smart-Nultra-pure water system and used for all of the experiments. All other chemicals were analytical grade and used without further purification. 50 mM of NaAc-HAc (pH 5.0) was used for DNA and miRNA dissolution, 50.0 mM of tris-HCl (pH 7.4, with 50 mM of NaCl) was used for miRNA-21 detection.

UV-vis absorption spectra of (+)AuNPs were carried out on an evolution 60 spectrophotometer (Thermo Fisher Corporation, USA). The size distribution of (+)AuNPs was characterized by transmission electron microscope (TEM, JEM-2010HR, Japan). The zeta potential analysis of (+)AuNPs was investigated using particle size analyzer (MS2000, England).

### Preparation and characterization

(+)AuNPs were prepared according to our previous method^65^. Firstly, 15 mL of HAuCl_4_ (1.0 mM) and 2 mL of CTAB (10 mM) were mixed and stirred for 15 min. Subsequently, 2 mL of NaBH_4_ (100 mM) was added to the mixed solution and kept stirring for another 8 min. After the solution color changed to orange red and without changing within 15 min, the solution was filtered and stored in a refrigerator at 4 °C before use. The average size of such (+)AuNPs was about 4 nm estimated from TEM images (Fig. 3A(insert a)). Meantime, the zeta potential of such nanoparticles monitored form DLS were positively charged (Fig. 3B(a)).

To carry out the gel electrophoresis, four samples including target miRNA-21 (1.0 μM), H_1_ (1.0 μM), H_1_ and H_2_ (1.0 μM, 1:1 ratio) mixture in the absence and presence of miRNA-21 (1.0 μM) were prepared. After that, 4 μL of each DNA samples were loaded into the lanes and performed at a constant potential of 65 V for 45 min on a 1.5 wt% agarose gel by using 1.0×TAE as running buffer. Then, gels were photographed by gel image system under UV light after Stains-All staining by ethidium bromide (EB) solution for 15 min.

### Visual detection of miRNA-21

H_1_ and H_2_ were firstly mixed and heated to 95 °C for 5 min, followed by cooling to room temperature gradually, and then they were stored at 4 °C prior to use. For miRNA-21 detection, different concentrations of target miRNA-21 samples were mixed with 10 μL of H_1_ and H_2_ mixture (300 nM, 1:1 ratio) separately, followed by incubating at 37 °C for 1 h to proceed the HCR. After that, 100 μL of (+)AuNPs (10.6 nM) were added into above solutions respectively and incubated for another 25 min. Due to the electrostatic adsorption of long-range DNA polymers with (+)AuNPs, (+)AuNPs precipitation appeared. Finally, the concentrations of (+)AuNPs in supernatant of each above mixed solutions were collected and detected by using UV-vis characterization.

### Visual detection of miRNA-21 in real samples

To evaluate the application of the sensor for miRNA-21 detection in real samples, human serum samples were collected from Xuzhou Central Hospital and diluted for 10 times with PBS buffer before using. Then, 50 pM, 500 pM and 5.0 nM of miRNA-21 samples were mixed with 10 μL of H_1_ and H_2_ mixture (300 nM, 1:1 ratio) separately, and then added them to human serum samples followed by incubating at 37 °C for 1 h to proceed the HCR. After that, 100 μL of (+)AuNPs (10.6 nM) were added into above solutions respectively and incubated for another 25 min. Finally, the concentrations of (+)AuNPs in supernatant of each above mixed solutions were collected and detected by using UV-vis characterization, and the concentration of miRNA was found from the calibration curve.

### Cell culture and total RNA extraction

Three types of human cancer cell lines including human breast cancer cell line (MCF-7), human cervical carcinoma cell line (HeLa) and human mammary epithelial cell line (MCF-10A) were cultured in Dulbecco’s Modified Eagle Medium (DMEM) supplemented with 15% fetal bovine serum (FBS), 100 U/mL penicillin and 100 g/mL streptomycin at 37 °C in a humidified atmosphere containing 5% CO_2_. The cellular extracts were prepared according to the literature method^64^.

## Additional Information

**How to cite this article**: Miao, X. *et al*. Sensitive detection of miRNA by using hybridization chain reaction coupled with positively charged gold nanoparticles. *Sci. Rep.* **6**, 32358; doi: 10.1038/srep32358 (2016).

## Figures and Tables

**Table 1:** Comparison of our method to other nanoparticle-based optical miRNA detection methods.

Material	Linear range	LOD	Ref.
AuNPs	20 pM to 1 nM	16 pM	18
Graphene/AuNPs	10 nM to 0.98 μM	3.2 nM	61
Graphene oxide	20 pM to 1 nM	9 pM	62
AuNPs	2.0 fM and 1.0 pM	1.0 fM	16
Gold nanoplasmonic particles	1.0 pM to 10 μM	—	63
(+)AuNPs	20 pM to 10 nM	6.8 pM	Our work

**Table 2:** Detection of miRNA-21 in human serum samples (n = 3).

Sample	Added	Found	Recovery (%)	RSD (%)
1	50 pM	49.6 pM	99.2	4.63
2	500 pM	506.3 pM	101.3	3.86
3	5.0 nM	4.89 nM	97.8	3.38

**Table 3:** DNA and RNA sequences.

Name	Sequence (5′–3′)
H_1_	TCAACATCAGTCTGATAAGCTACAAAGTAGTCTAGGATTCGGCGTG
H_2_	TAGCTTATCAGACTGATGTTGACACGCCGAATCCTAGACTACTTTG
MiRNA-21	UAGCUUAUCAGACUGAUGUUGA
Mismatch 1	UAGCUUAUCAGACUGACGUUGA
Mismatch 2	UAGCGUAACAGACUGACGUUGA
